# Author Correction: Mechanically tunable conductive interpenetrating network hydrogels that mimic the elastic moduli of biological tissue

**DOI:** 10.1038/s41467-018-07487-1

**Published:** 2018-11-23

**Authors:** Vivian R. Feig, Helen Tran, Minah Lee, Zhenan Bao

**Affiliations:** 1 Department of Material Science and Engineering, Stanford University, 443 Via Ortega, Room 307, Stanford, CA 94305 USA; 2 Department of Chemical Engineering, Stanford University, 443 Via Ortega, Room 307, Stanford, CA 94305 USA

Correction to: *Nature Communications*; 10.1038/s41467-018-05222-4; published online: 16 July 2018

The original version of this Article contained an error in the second sentence of the ‘Materials’ section of the Methods, which incorrectly read ‘PEDOT:PSS synthesized by Orgacon (739324 Aldrich, MDL MFCD07371079) was purchased as a surfactant-free aqueous dispersion with 1.1 wt% solid content.’ The correct version replaces this sentence with ‘PEDOT:PSS Orgacon ICP 1050 was provided by Agfa as a surfactant-free aqueous dispersion with 1.1 wt% solid content.’ This has been corrected in both the PDF and HTML versions of the Article.

