# Supplementary material for: Mechanically tunable conductive interpenetrating network hydrogels that mimic the elastic moduli of biological tissue
Source: Nat Commun. 2018 Jul 16;9:2740. doi: 10.1038/s41467-018-05222-4 (PMC6048132; doi:10.1038/s41467-018-05222-4)
Supplement: Supplementary file 1 — Supplementary Information [file 41467_2018_5222_MOESM1_ESM.pdf]

Supplementary Information

Mechanically tunable conductive interpenetrating network hydrogels that mimic the elastic moduli of biological tissue

Feig et al.

Supplementary Figure 1

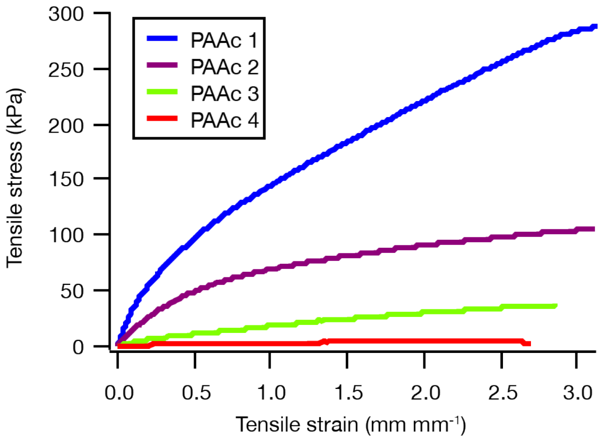

Supplementary Figure 1. Tensile elongation behavior of polyacrylic acid hydrogels. The modulus of polyacrylic acid hydrogels, as indicated by the initial slope of the stress/strain curve, can be easily tuned by varying the monomer concentration and the bisacrylamide to acrylic acid ratio.

Supplementary Figure 2

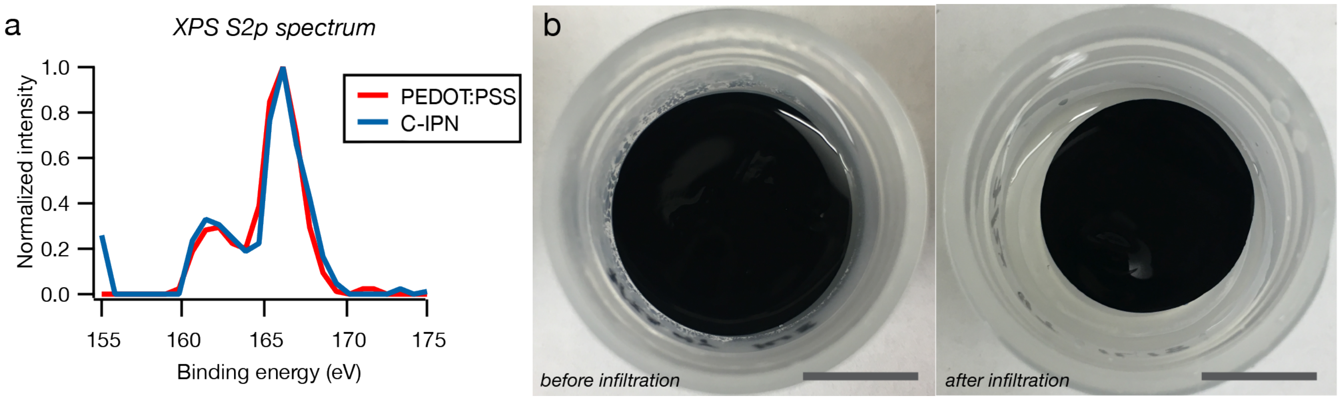

Supplementary Figure 2. Influence of secondary network infiltration on chemical and physical properties of PEDOT:PSS hydrogels. (a) XPS spectra at the binding energy of S2P. The peaks correspond to sulfur on PEDOT and PSS, respectively. The overlap between S2P peaks in PEDOT:PSS alone and in C-IPN indicates that the process of infiltrating PEDOT:PSS with PAAc does not significant affect the chemical structure or composition of PEDOT:PSS. (b) The hydrogel appears to shrink slightly after infiltrating PEDOT:PSS hydrogels with acrylic acid and bisacrylamide. Scale bars are 10 mm.

Supplementary Figure 3

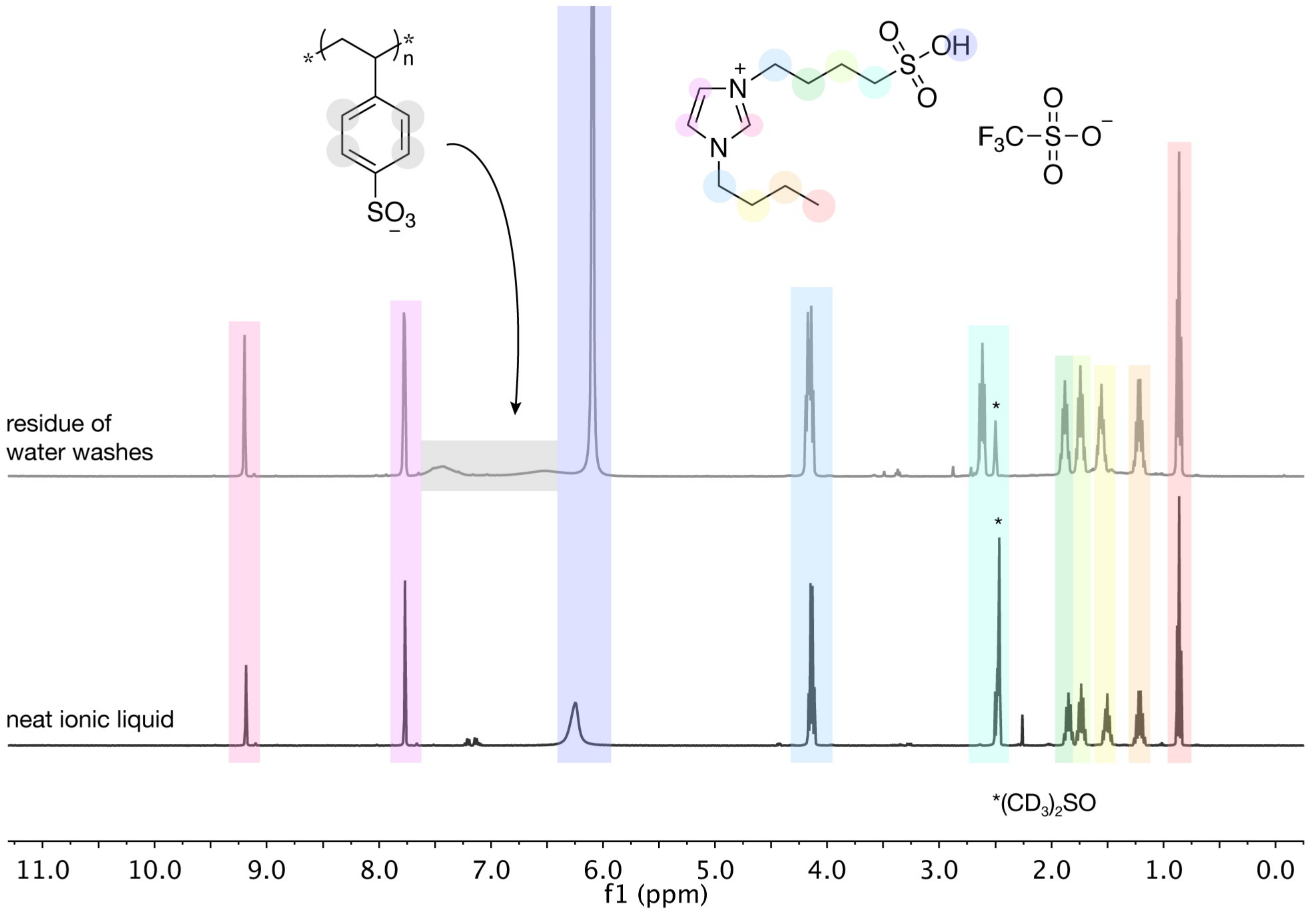

Supplementary Figure 3. H-NMR spectrum comparing neat ionic liquid, with the solution salvaged from washing PEDOT:PSS hydrogels with DI water. The excellent overlap between the two spectra indicates that a significant amount of ionic liquid can be washed away from simply exchanging PEDOT:PSS in DI water. ^{1}H NMR (400 MHz, DMSO): δ 9.20 (s, 1H), 7.78 (m, 2H), 7.70-6.25 (only in residue wash, m, 4H), 6.61 (s, 1H), 4.14 (m, 4H), 2.62 (m, 2H), 1.88 (m, 2H), 1.74 (m, 2H), 1.55 (m, 2H), 1.21 (m, 2H), 0.86 (m, 3H).

Supplementary Figure 4

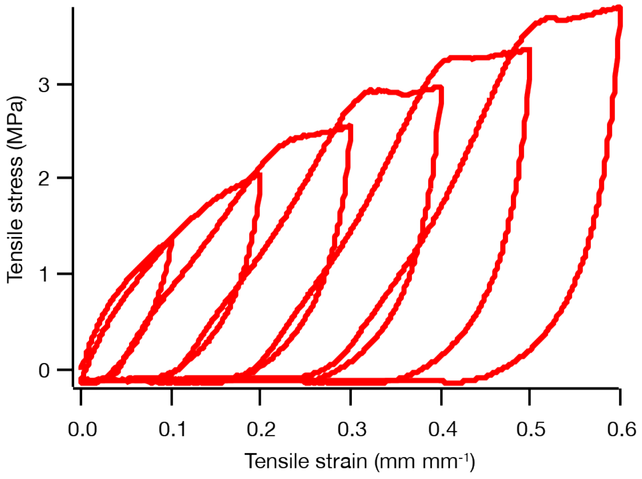

Supplementary Figure 4. Tensile cycling of ILASTEC, a stretchable conductor developed in our group consisting of PEDOT:PSS and an ionic liquid.[1] While highly stretchable, the conductor has poor elasticity, as evidenced by1 the large hysteresis in its cyclic stress/strain curves.

Supplementary Figure 5

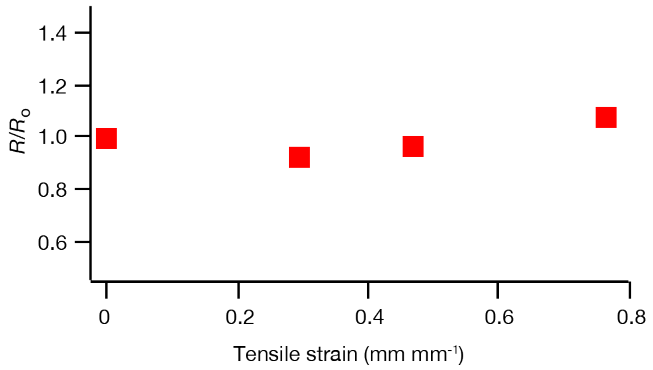

Supplementary Figure 5. Resistance change while stretching, with resistance measured using a 4-point probe. Resistance values were manually recorded while stretching C-IPN gels to various lengths.

Supplementary Table 1

Hydrogel system	Conductivity ($S\ m^{-1}$)	Modulus (kPa)	PEDOT:PSS content (wt%)	Reference
PAAc/PEDOT:PSS	0.01-0.1	50	18.4	14
PAAm/PEDOT:PSS	0.26	300	30.6	13
PNAGA/PEDOT:PSS	0.2-2.2	30-110	1-10	15
C-IPN	12.7-23.7	8-374	1.1	This work

Supplementary Table 1. Comparison between C-IPN with other stretchable PEDOT:PSS-based hydrogels in the literature.

Supplementary Figure 6

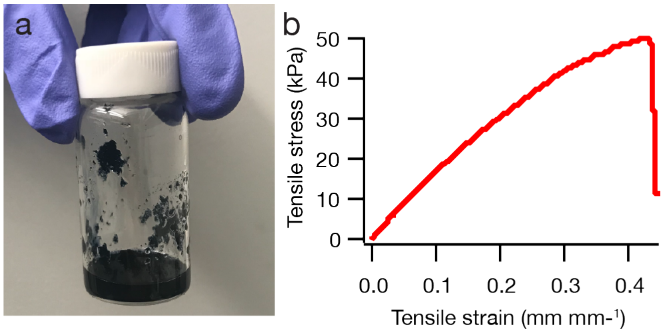

c

sample	conductivity ($S\ m^{-1}$)	modulus (kPa)	strain at break (%)
C-IPN 1	23.1 ± 5.6	374	121
PAAc 1 / PEDOT blend	0.21	177	43

Supplementary Figure 6. Comparison of C-IPN hydrogels to gels formed by direct blending. (a) Blending PEDOT:PSS with acrylic acid monomers caused PEDOT:PSS to rapidly crash out of solution. (b) After polymerizing the blend, the material was still stretchable. (c) However, the conductivity, modulus, and strain at break were all significantly worse than the equivalent C-IPN formulation.

Supplementary Figure 7

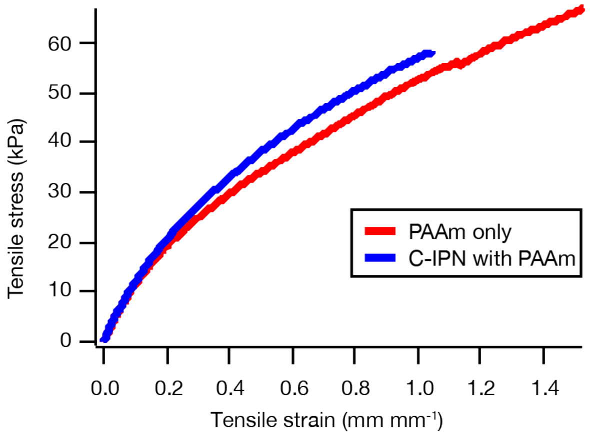

Supplementary Figure 7. C-IPN could similarly be fabricated with polyacrylamide (PAAm). As with C-IPN made with PAAc, the C-IPN with PAAm had similar mechanical properties as PAAm on its own.

Supplementary Figure 8

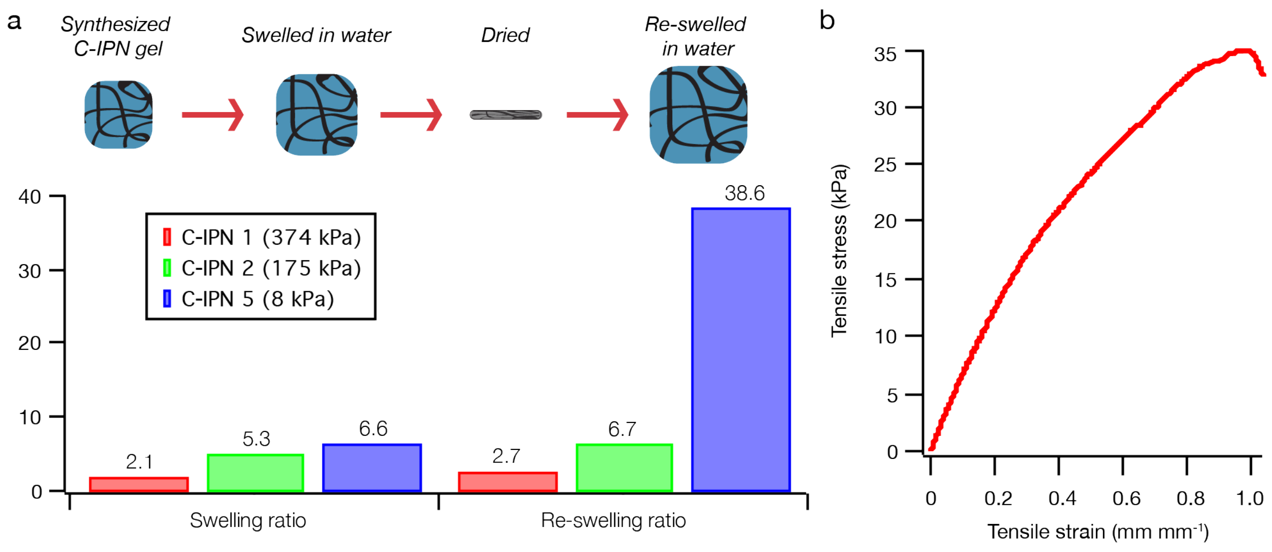

Supplementary Figure 8. Swelling behavior of C-IPN hydrogels. (a) C-IPN hydrogels continue to swell when immersed in DI water. The swelling ratio is the ratio of the final weight of the swollen hydrogel to its as-synthesized weight. Furthermore, C-IPN hydrogels are capable of re-swelling to greater than their original weight after they have been dried. The re-swelling ratio is the ratio of the final weight of the re-swollen hydrogel to its as-synthesized weight. Hydrogels fabricated with lower initial solid content have a lower modulus, and also swell and re-swell to a larger extent than those fabricated with higher initial solid content. The softest gel, C-IPN 5, can re-swell to over 38 times its original weight when it is immersed in water after drying. (b) Tensile elongation data for a C-IPN3 gel (original modulus of 99 kPa) that has been dried and re-swelled in DI water. The re-swelled gel retains its high stretchability (>100% strain) and low modulus (70 kPa).

Supplementary References

1. Wang, Y. *et al*. A highly stretchable, transparent, and conductive polymer. *Sci. Adv.* **3,** (2017).
